# Benzoate Drugs for Traumatic Brain Injury

**DOI:** 10.33140/jcei.11.01.02

**Published:** 2026-03-14

**Authors:** Swarupa Pahan, Kalipada Pahan

**Affiliations:** 1 Division of Research and Development, Jesse Brown Veterans Affairs Medical Center, Chicago, USA; 2 Department of Neurological Sciences, Rush University Medical Center, Chicago, USA

**Keywords:** Traumatic Brain Injury, Sodium Benzoate, Glyceryl Tribenzoate, Glial Activation, Neurodegeneration, Oxidative Stress, Cognitive Dysfunction, Neuroprotection, Drug Repurposing

## Abstract

Traumatic brain injury (TBI) is an acute disorder with prolonged complexities that affirm to be the leading cause of deaths and disabilities in US. The pathophysiology of TBI comprises of multiple complex processes. An impactful insult results in tissue damage, neuroinflammation, impaired cerebral blood flow, BBB disruption, and demyelination, leading to neurodegeneration and functional impairments. There are no specific treatments for TBI. Recent studies highlight the potential efficacy of the following benzoates for attenuating TBI pathologies in rodents:

- Sodium benzoate, a metabolite of cinnamon, a widely used food additive and a Food and Drug Administration (FDA)-approved drug for glycine encephalopathy
- Glyceryl tribenzoate, FDA-approved flavoring element being used in food and food packing productions

In this review, we have made a genuine attempt to evaluate the significance of these two benzoates as prospective therapeutic options for TBI.

## 1. Introduction

Traumatic brain injury (TBI), also known as an intracranial injury, is caused by sudden jolt, bump or blow on the head due to fall, automobile crash, firearms, sports related injuries, and exposure to explosive at war [1,2]. Studies have shown that nearly half of the total TBI-related hospitalization is associated to fall and that firearm-related suicide is the most common cause of TBI-initiated death in US [3]. CDC report on TBI indicates that there were over 2.1 million emergency department visits for injuries from motor vehicle crashes and that about 41,000 people died in motor vehicle crashes in US in.2020. It has been reported that 5.3 million Americans are disabled of which 80,000 victims suffer from long term disability and about 70,000 deaths reported annually. TBI has significantly impacted the wellbeing of millions globally in all age groups, leading to temporary, permanent disabilities and untimely deaths [4]. To recoup with the devastating outcome of TBI, about $76.5 billion is attributed for care and loss of productivity every year. Studies show that the pathophysiology of TBI include neuroinflammation followed by neurodegeneration resulting in locomotive, cognitive dysfunctions, and neuronal deaths [5,6]. Regrettably, treatments available for TBI can only treat the symptoms to a certain extent and thereby only rest and medications like antianxiety, anticoagulant, anti-convulsant, antidepressants and muscle relaxants are prescribed for stabilization till surgery, which is evidently a perilous procedure. Therefore, there is an absolute need to investigate for a safe, nontoxic, conveniently administrable, and economic therapeutic agent to potently curb the devastating outcomes of TBI. In rodents, TBI like pathologies are induced by weight drop technique, controlled cortical impact (CCI), blast injury, and fluid percussion injury [7–10]. Studies show NaB, an FDA-approved treatment for urea cycle disorder and glycine encephalopathy in children, and GTB, a flavoring agent approved by Flavor and Extract Manufacturers Association (FEMA), can competently inhibit neuroinflammation and prevent neurodegeneration by improving locomotor and cognitive dysfunctions in CCI-induced mouse model of TBI [11,12]. Our investigation focuses on the neuroprotective efficacy of these two benzoates and evaluates the potential therapeutic possibilities for TBI treatment.

## 2. Clinical symptoms of TBI

TBI is an aggressive and amalgamated disease process that leads to impairment of different body functions or even death in all age group of the population, especially in older adults with worse functional outcomes and higher mortality [1,2]. TBI can be classified by the nature of the injury, penetrating (open) and non-penetrating (closed) TBI. Penetrating TBI is caused by sharp weapon or bullet entering the skull and affecting the part of the brain that encounters the insult. Non- penetrating (closed) TBI usually is caused by an external insult on the head acting on the whole brain exhibiting devastating clinical symptoms [13]. TBI can also be classified depending on the severity of the impact of the insult ranging from mild, moderate to severe. Clinical symptoms of mild TBI are marked by brief loss of consciousness, headache, confusion, dizziness, blurred vision and ear ringing, bad taste in mouth, fatigue, mood changes, sleep pattern trouble, and memory issues [7]. Moderate to severe TBI symptoms are characterized by headache getting worse, repeated vomiting and nausea, convulsion, seizures, numbness, weakness of arms and legs, loss of coordination, increase in confusion, restlessness and agitation, and not being able to wake up from sleep or coma and death.

### 2.1. Cognitive Deficit

It is caused by impairments of the network of neurons of the temporal and frontal lobes of the brain that primarily affect learning, memory, perception, and problem-solving abilities. Cognitive dysfunction is one of the problematic outcomes among TBI survivors [14]. Similar clinical symptoms of cognitive deficits are observed in one of the most prevalent neurodegenerative disease, Alzheimer’s disease (AD) [15]. Clinical studies on postmortem brains of TBI victims have shown an increased level of hyperphosphorylated tau (P-tau) and amyloid beta and TDP 43 deposits, indicating AD-like pathologies in TBI condition [16]. Studies also indicate that older adults with a history of moderate TBI are at 2.5 times greater risk of developing AD than seniors with no history of TBI and that persons with a history of severe TBI have 4.5 times greater AD risk than non-TBI population [16].

### 3.2. Psychological Problems

Mood disturbance, depression and anxiety are neuropsychiatric disorders regulated by frontal cortex, basal ganglia and temporal lobes of the brain that significantly impact the behavioral and emotional aspect and affect recovery, functional ability, and daily life activities of the concern individual [17]. TBI survivors experience neuropsychiatric dysfunctions at a much higher rate than general population. Depression is a common mental disorder characterized by feeling of sadness and hopelessness, which persist and eventually interfere with daily normal activities of the affected individual. TBI and depression have a close association. Studies show about 6–77% of TBI patients experience depressive disorder, among which 25–50% diagnosed with first-year post-TBI depressive disorder and 26–64% have life-time risk of depression development [18]. Similarly, anxiety is also a TBI consequence that affects about 20% of TBI patients within one year of injury [19]. It has been shown that TBI individuals are 1.9 times more likely to develop anxiety symptoms as compared to normal individuals [19].

### 3.3. Post-Traumatic Stress Disorder (PTSD)

PTSD is a mental disorder caused by experiencing or witnessing terrifying incidents. It is characterized by dysphoric mood, anxiety, sleep disturbances, irritability, anger, poor concentration, fatigue, and upsetting memory of the terrible event. Epidemiological data confirms that PTSD development is significantly prominent among mild TBI survivors [20]. Moreover, rate of PTSD following a brain injury is higher in military service men than in civilians due to their prolonged exposure to combat. About 35% returning military with a mild brain injury experience PTSD [21]. Proper diagnosis and treatment are needed for managing these conditions.

### 3.4. Locomotor Impairment

Across the spectrum of TBI from mild to moderate to severe, patients suffer from locomotor impairment. In some TBI patients, shearing of axons and excess neuroinflammation initiate the loss of communication of signals hindering coordination and resulting in tremor, akinesia, and postural instability, triad symptoms that are hallmarks of Parkinson’s disease (PD), the most common neurodegenerative movement disorder caused by the demise of dopaminergic neurons present in the substantia nigra compacta region of the midbrain. It has been shown that TBI is associated with a risk for Lewy body accumulation and parkinsonism [22,23]. LRRK2 is connected with late onset of PD and recent studies indicate the activation of LRRK2 in TBI mice [24]. Studies also demonstrate the upregulation of amyloid precursor protein (APP), hyperphosphorylation Tau and TAR DNA-binding protein 43 (TDP-43) in TBI patients, which are closely linked to PD. It has been also shown that TBI leads to PD-like pathologies in mice [23,25]. According to Delic et al., veterans with a history of mild TBI are at 56% higher risk for developing PD later in life and the PD risk grows with increase in TBI severity. Therefore, TBI is definitely a risk factor for PD [23].

## 4. Pathological hallmarks of TBI

Progression of TBI can be distinguished in 3 phases -acute, post-acute and chronic. While the acute phase initiates immediately upon insult to a week, the post-acute phase lasts from 1 week to 1 month. On the other hand, the chronic phase lasts from 1 month to years. Here, we will describe key pathological features that are present in different phases of TBI.

### 4.1. Neuroinflammation

Immediately after an insult, the brain’s immune system gets stimulated and initiates neuroinflammation to combat and heal. Therefore, neuroinflammation has a beneficial window and is not bad all the time. However, this essential process sometimes prolongs, leading to uncontrolled with upregulated production of proinflammatory molecules like TNFα, IL-6, IL-1β, nitric oxide (NO), etc. [26–32]. Then the chronic unrestrained neuroinflammation becomes detrimental for the brain. It has been reported that TNFα is capable of promoting the actin stress fiber formation and downregulating the tight junction protein occludin, resulting in retraction of endothelial cells and creation of gaps in the BBB [33]. This allows the compromised BBB to facilitate the infiltration of different cytotoxins into the brain causing neurodegenerative damage.

### 4.2. Demyelination

Although previous studies on TBI stressed on pathological modifications in neuronal cells within the gray matter, recent studies have highlighted the equal importance of white matter integrity in long-term recovery from TBI-related damages [34]. Demyelination is an important feature of white matter injury that is characterized by the loss of the myelin sheath [35,36]. Oligodendrocytes are the primary cells responsible for the generation and maintenance of myelin sheath under normal conditions and for remyelination after axonal damage [37–39]. However, the loss of oligodendrocytes is known to be a significant factor underlying demyelination after CNS injury as myelinating oligodendrocytes are highly vulnerable to ischemic or traumatic insults [40–42]. Accordingly, using a large-scale cohort study design, Kang and Lin have examined the risk for multiple sclerosis (MS) following a TBI and found increased risk of MS after TBI [43].

### 4.3. Axonal Injury

In white matter tracts, axons are encapsulated by oligodendrocyte-generated myelin. The axon- derived signals are required for the proliferation, migration, survival, and differentiation of oligodendrocytes [44,45]. In addition, oligodendrocytes are also actively involved in detecting axonal energy needs and preserving axonal integrity [46]. Therefore, once an axon is demyelinated, its ability to transmit action potentials and its energy supply are severely impaired, causing the exposed nerve fiber to be highly susceptible to degeneration during TBI. This hinderance of communication results in loss of physical and mental coordination or disabilities among TBI patients. Although the etiology of MS is unknown, in many MS patients, demyelination of axons is seen [47]. A systematic review and meta-analysis have delineated that the risk of MS increases among people with a history of head trauma [48].

### 4.4. Intracerebral Hematoma and Edema

Intracerebral hematomas and edema are the most damaging outcome of TBI [49]. Intracerebral hematoma is the accumulation of blood within the brain due to coalescence of contusion upon TBI. This results in increased intracranial pressure leading to brain damage, unconsciousness or even death. Edema is the swelling caused by fluid leak in blood vessel into nearby tissues due to the rapture of the vessels during the brain injury. Hematoma and edema result in brain compression and increase in ICP and BBB disruption, subsequently developing enlarge lesion cavities in the temporal lobes. This is probably the leading cause to mortality and mobility for TBI patients.

### 4.5. Oxidative Stress

Oxidative stress is caused by an imbalance between the reactive oxygen species (ROS) and the antioxidant defense system in the cells. The overproduction of ROS\ indulges in harmful chains of oxidation reactions resulting in the breakdown of cells and leading to serious neurological diseases like MS, AD, PD, HD, etc. [50–53]. Studies have shown the involvement of oxidative stress in the pathogenesis of TBI [54]. The level of CSF malondialdehyde (MDA), oxidative stress marker, is elevated within 2–3 hours of TBI and remain elevated for several days post injury [55]. Moreover, the activity of antioxidant defense enzyme superoxide dismutase (SOD) decreases 24 hours post TBI and remain so 7 days post severe TBI [54]. Moreover, oxidative stress is known to trigger the production of proinflammatory molecules via activation of NF-κB [56]. However, studies have shown that NaB treatment can reduce LPS-induced production of ROS in mouse microglia [57]. NaB also inhibits ROS production from microglia stimulated by fibrillar amyloid-β (an etiological reagent for AD) and 1 methyl-4-phenylpyridinium ion (a Parkinsonian toxin) [57]. Small G protein p21^rac^ is an important member of NADPH oxidase, a five-subunit enzyme complex that catalyzes the production of superoxide radical [58]. NaB inhibits the activation of p21^rac^ via reducing the geranylation pathway and thereby decreases the production of ROS from activated microglia [57]. Oral administration of NaB also exhibits antioxidant effect *in vivo* in the brain of 5XFAD mouse model of AD [57]. Following the same mechanism, NaB treatment may also reduce oxidative stress in the hippocampus under TBI condition.

## 5. Sodium benzoate in TBI

NaB is a metabolite of cinnamon, a household spice and flavoring agent [59,60]. NaB is also a food-additive and an FDA-approved drug against urea cycle disorders and glycine encephalopathy [61,62]. CCI-induced mouse model is a reliable model to showcase the symptoms of TBI [10]. Recent study has highlighted the beneficial effect of NaB in CCI-induced TBI mice [12].

### 5.1. Inhibition of Glial Activation

**Figure 1:**
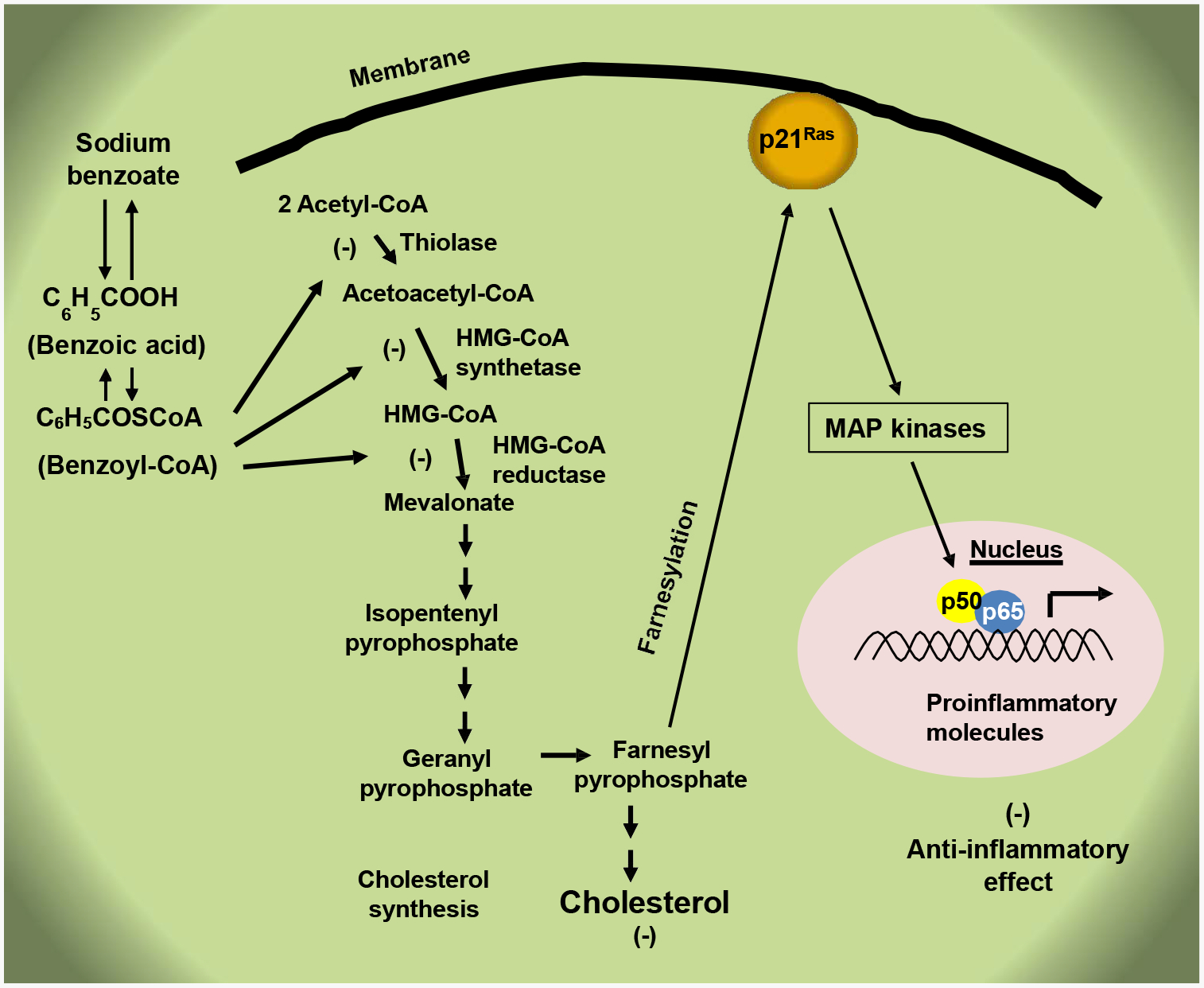
Anti-inflammatory effect of NaB. Cholesterol is synthesized within the cells from acetyl-CoA via multiple steps. NaB forms benzoyl-CoA, which competitively inhibits the first three steps of the cholesterol biosynthesis pathway catalyzed by thiolase, HMG-CoA synthetase and HMG-CoA reductase, respectively to lower the level of farnesyl pyrophosphate. The small G protein p21^ras^ gets attached to the membrane and becomes activated upon farnesylation by farnesyl pyrophosphate. Therefore, by lowering the level of farnesyl pyrophosphate, NaB inhibits the activation of p21^ras^ and associated mitogen-activated protein (MAP) kinase pathway to lower the activation of classical NF-κB p65:p50 heterodimer and the expression of proinflammatory molecules, leading to anti-inflammation.

Upon brain injury, glial cells like astroglia and microglia are activated to express higher levels of GFAP and Iba-1, respectively. Moreover, activated glial cells produce a wide variety of proinflammatory molecules like TNFα, IL-1β, IL-6, IL-8, macrophage inflammatory protein-1 alpha (MIP-1α), NO, etc., which could ultimately lead to neuroinflammation and neurodegeneration [31,63–66]. However, oral NaB treatment leads to decrease in both GFAP positive astrocytes and Iba-1 positive microglia in the cortex and hippocampus of CCI-induced TBI mice [12]. Activated glial cells also express elevated levels of iNOS for the induced production of NO, which sometimes leads to nitrosative stress [31,65,67]. It is encouraging to see that NaB treatment results in the suppression of iNOS in astroglia and microglia *in vivo* in the brain of TBI mice [12]. It has been described that NaB inhibits the expression of proinflammatory molecules in activated glial cells via suppression of farnesylation of p21^ras^ and activation of NF- κB pathway (Figure 1) [68].

### 5.2. Reduction of Lesion Volume

TBI often causes enlarged lesion cavity due to the impact of the external insult, which can culminate into serious immunological and neurological disorders with fatal consequences. Considering segmentation of the brain, diffusion, and the damage regions, the lesion region in the brain tissue is usually estimated. The area of the damaged portion is estimated across each slice of MRI after the segmentation followed by estimation of the combined volume of damage through 3D reconstruction. Accordingly, in CCI-induced TBI mice, a typical lesion with enlarged cavity originating from the cortex through the hippocampus and connecting to the lateral ventricle is seen.

Consistent to the suppression of glial activation, oral NaB is capable of reducing the lesion size in the hippocampal region of the brain in TBI mice as detected by the Cavalier stereological techniques [12].

### 5.3. Protection of Memory and Learning

Learning and memory is regulated by the hippocampal region of the brain and the dysfunction of memory and learning is likely to be a major drawback for TBI survivors for the rest of their lives [69]. It has been found that NaB is capable of upregulating plasticity-related molecules, stimulating NMDA- and AMPA-sensitive calcium influx and increasing the spine density of cultured hippocampal neurons [70]. As a result, NaB has been reported to convert poor learning mice to good learning ones [70]. While Barnes maze and T-maze are used for monitoring spatial learning and memory in mice, novel object recognition (NOR) test is used to evaluate short-term memory [71,72]. AD is the most common memory disorder and it has been found that daily oral NaB feeding for 1 month improves memory and learning in 5XFAD mouse model of AD [57]. Similar to 5XFAD mice, the CCI-induced TBI mice also exhibit deficient spatial learning and memory and short-term memory as compared to sham mice [12]. Daily NaB treatment recovers both spatial learning and memory and short-term memory in CCI-induced TBI mice, indicating that NaB is capable of improving hippocampal functions in TBI condition [12].

### 5.4. Reduction of Amyloid Plaques

AD is the most common neurodegenerative disorder causing dementia among the older population around the world [73,74]. Pathologically, AD is characterized by neurofibrillary tangles and neuritic plaques comprising of aggregated Tau and Aβ, respectively [75–78]. It has been shown that oral NaB treatment decreases amyloid plaques from the hippocampus of 5XFAD mouse model of AD [57]. Although amyloid plaques are typically associated to AD and aging, it has been reported that TBI can lead to the formation of amyloid plaques, indicating a possible reason for increased risk of developing AD among TBI individuals [79]. Therefore, by reducing the formation of plaques, NaB may be also beneficial for TBI patients.

### 5.5. Restoration of Dopamine Neurons and Dopamine

Dopamine (DA), a neurotransmitter crucial for various brain functions, is produced by dopaminergic neurons from tyrosine via tyrosine hydroxylase (TH) [80]. Although PD, the most common neurodegenerative movement disorder, is caused by loss of DA, studies have shown that TBI can also cause DA loss leading to neuropsychiatric symptoms and cognitive impairments [81]. However, it has been shown that NaB can increase the mRNA and protein expression of TH and stimulates the production of DA from dopaminergic neuronal cells [82]. Mechanistically, NaB stimulates the transcription of *TH* in dopaminergic neurons via the activation cAMP response element-binding [82]. Oral feeding of NaB also increases the expression of TH in the nigra, upregulates striatal DA, and improves locomotor activities in normal C57/BL6 and aged A53T-α- syn transgenic mice [82]. Therefore, NaB may also increase the level of DA in the brain of TBI patients for improving movement, reward, cognition, and mood.

### 5.6. Protection of Locomotor Activities

**Figure 2:**
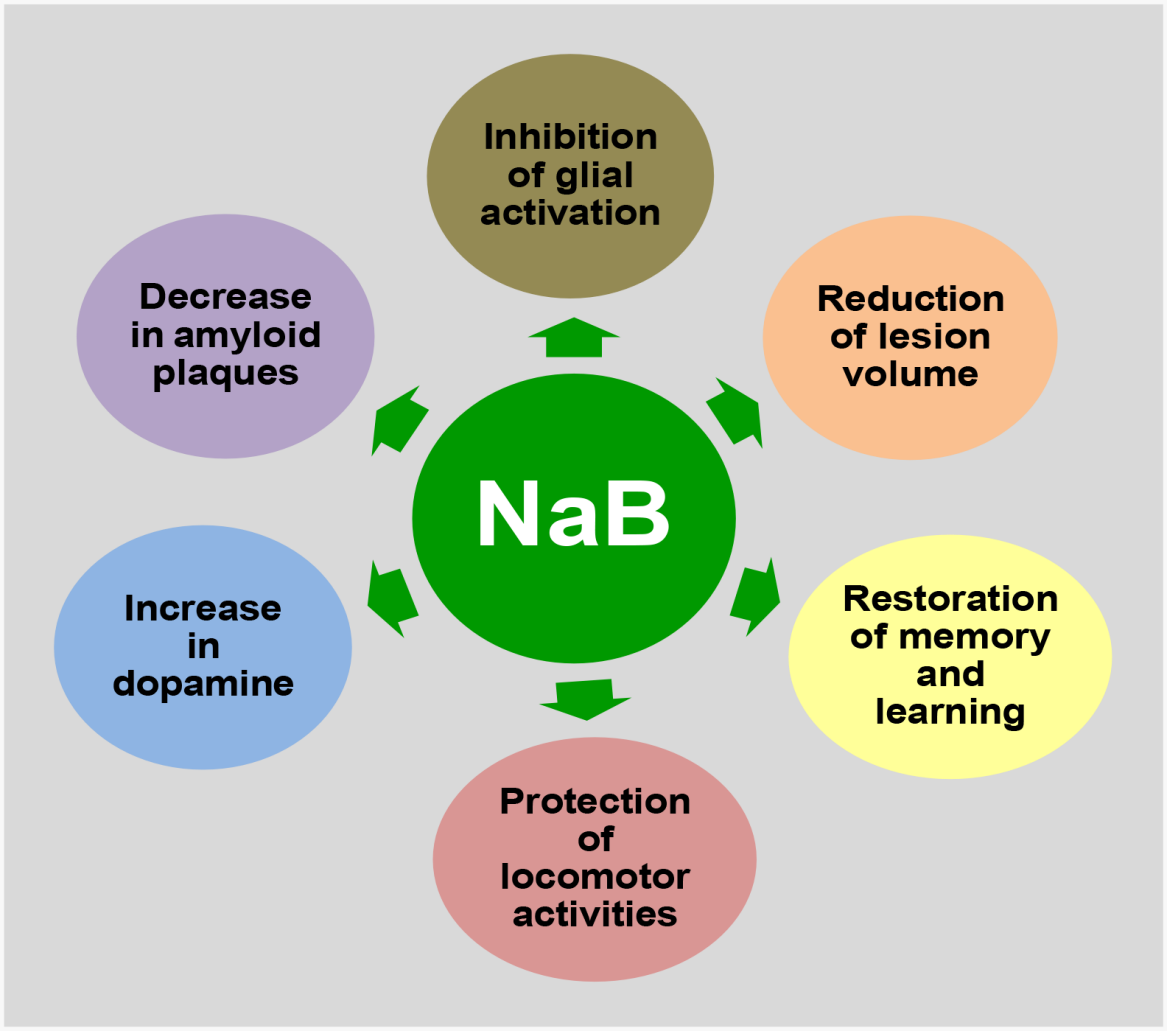
Anti-TBI functions of NaB. In one hand, NaB inhibits glial inflammation, reduces amyloid plaques and decreases lesion volume, and on the other, increases the production of dopamine, upregulates memory and learning and improves locomotor activities to exhibit neuroprotective effects

Following TBI, locomotor problems, particularly gait and balance disturbances, are common. This locomotive dysfunction in TBI patients is manifested by weakness as well as loss of neurons ( ). External insults can cause neuronal structural damage, resulting in neuronal loss and locomotor dysfunction in TBI. However, as compared to untreated TBI mice, NaB treatment leads to significant improvement in locomotor activities such as distance traveled, velocity, center frequency, rearing behavior, rotarod performance, gait behavior, and grid performance in TBI mice [12]. In addition to TBI, oral NaB treatment is capable of improving locomotor activities in mouse models of PD, MS and Lewy body dementia [83–85]. Therefore, NaB may exhibit beneficial effects for TBI via suppression of pro-TBI as well as induction of anti-TBI pathways (Figure 2).

## 6. Glyceryl tribenzoate (GTB) in TBI

GTB is a flavoring reagent used in food and packaging industry [86]. Here, we will discuss neuroprotective effects of GTB in TBI mice.

### 6.1. Attenuation of Neuroinflammation

Since neuroinflammation is an important pathological feature of TBI, a prospective drug should attenuate neuroinflammation in order to be successful in TBI. While astroglial and microglial activation is evident in the brain of TBI mice, oral administration of GTB reduces the number of activated astroglia and microglia in TBI mice [11]. GTB treatment also decreases the level of iNOS protein in the brain and reduces the number of iNOS-expressing astroglia and microglia in hippocampus and cortex of TBI mice [11].

### 6.2. Reduction of Axonal Damage

It is established that combination of mechanical insult and resultant neuroinflammation have a remarkable impact on the synaptic structure and function, leading to synaptic dysfunction [87]. It is also known that several plasticity-related proteins such as PSD-95, NR2A and GluR1 are involved in synapse development and maturation [76,88,89]. Studies show that CCI-induced TBI decreases the levels of PSD-95, NR2A and GluR1 in the hippocampus region of the TBI mice compared to sham control [11]. Interestingly, oral GTB treatment leads to upregulation and/or normalization of PSD-95, NR2A and GluR1 in the hippocampus of TBI mice [11]. Therefore, GTB treatment can evidently contribute to synapse development and maturation in the hippocampus of TBI mice.

### 6.3. Decrease in Lesion Volume

Lesion volume is an important measure used to evaluate the degree and severity of injury in TBI brains. The Cavalieri stereological technique is used to determine the lesion volume in rodents with TBI [11,12,90]. In a study with CCI-induced TBI in mice, a typical lesion with distend cavity originating from cortex through hippocampus and involving to the lateral ventricle is seen. However, daily oral treatment with GTB leads to decrease in total lesion volume in the whole hemisphere of TBI mice as compared to untreated TBI mice [11]. Therefore, GTB is capable of reducing lesion volume in TBI mice.

### 6.4. Improvement of Cognitive Functions

Cognitive deficiency is common among many TBI survivors and it has been reported that the cognitive defects in TBI is probably due to impaired synaptic alterations [91,92]. Since GTB treatment attenuates neuroinflammation, decreases lesion volume and restores synaptic maturation in TBI mice, the study also investigates the effect of GTB on cognitive functions and reports significant improvement in spatial memory and learning and short-term memory in TBI mice after GTB treatment [11]. Therefore, this flavoring agent may be considered for improving cognitive functions.

### 6.5. Restoration of Locomotor Activities

In many individuals, TBI can disturb balance and coordination, thus increasing the risk of falls, especially in the elderly. Therefore, improving locomotor function after a TBI may have a remarkable influence on recovery journey and overall well-being of a patient. It has been reported that treatment of CCI-induced TBI mice with oral GTB leads to significant increase in distance travelled, velocity, center frequency, rearing, rotarod performance, beam walking, and grid runway as compared to untreated TBI mice [11]. Therefore, oral GTB is capable of restoring and/or improving open field behavior, motor coordination and balance activity, and gait behavior in a mouse model of TBI.

## 7. Conclusion

Currently, no therapies are available that can certainly halt the progression of TBI. Although some treatments are there for taking care of blood clots, muscle spasms, anxiety, depression, and mood swings, many of the available drugs simply display symptomatic support. Moreover, the available drugs exhibit a number of undesirable effects. Therefore, development of effective treatment options for TBI is an important area of research. There are various advantages of NaB and GTB over available anti-TBI therapies. First, NaB has therapeutical importance in treating urea cycle disorders and glycine encephalopathy. GTB is also an FDA-permitted flavoring constituent for being used in food and food packaging businesses. Second, both NaB and GTB can be taken orally, the least painful route of drug treatment. Consistent with that found in mouse models MS, PD, AD, and Lewy body dementia, NaB reduces glial activation in the hippocampus and protects memory and learning in TBI mice [57,83–85]. Neuroprotective effects of GTB have also been reported in mouse models of Huntington disease and multiple sclerosis and a monkey model of Parkinson’s disease [93–95]. Accordingly, oral GTB also exhibits beneficial effects in TBI mice [11]. Therefore, these benzoate drugs (NaB and GTB) may be considered for repurposing as therapeutic agents in TBI treatment.

## Data Availability

This is a review and the readers can access all the published article supporting the conclusions of this study through PubMed.
